# Identification and Structure–Activity Relationship of a Novel Dermatoxin-like Antimicrobial Peptide with Partial LPS-Mediated Membrane Interaction

**DOI:** 10.3390/molecules31162748

**Published:** 2026-08-07

**Authors:** Shiya Cheng, Yu Zai, Jiayi Peng, Jie Xiang, Xiaoling Chen, Chengbang Ma, Yangyang Jiang, Tao Wang, Tianbao Chen, Chris Shaw, Mei Zhou, Lei Wang

**Affiliations:** 1 Natural Drug Discovery Group, School of Pharmacy, Queen’s University Belfast, Belfast BT9 7BL, Northern Ireland, UK; scheng09@qub.ac.uk (S.C.); yzai01@jssnu.edu.cn (Y.Z.); jpeng08@qub.ac.uk (J.P.); jxiang@yzu.edu.cn (J.X.); xiaoling.chen@qub.ac.uk (X.C.); c.ma@qub.ac.uk (C.M.); t.chen@qub.ac.uk (T.C.); chris.shaw@qub.ac.uk (C.S.); m.zhou@qub.ac.uk (M.Z.); l.wang@qub.ac.uk (L.W.); 2 School of Life Sciences and Chemical Engineering, Jiangsu Second Normal University, Nanjing 210013, China; 3 School of Traditional Chinese Medicine, Faculty of Medicine, Yangzhou University, Yangzhou 225009, China; 4 Key Laboratory of the Jiangsu Higher Education Institutions for Integrated Traditional Chinese and Western Medicine in Senile Diseases Control, Yangzhou University, Yangzhou 225009, China

**Keywords:** antimicrobial peptides, dermatoxin, peptide truncation, structure–activity relationship, lipopolysaccharide interaction

## Abstract

The rapid emergence of antimicrobial resistance necessitates the development of novel antimicrobial agents with improved efficacy and selectivity. In this study, a dermatoxin-like peptide, dermatoxin-PD1, was identified from the skin secretion of *Pachymedusa dacnicolor*, and its structure–activity relationship was investigated through a rational truncation strategy based on predicted proteolytic cleavage sites. A series of truncated analogues was generated, among which a shortened peptide fragment (T1) retained potent antimicrobial activity, particularly against Gram-negative bacteria, whereas further truncation resulted in a marked loss of function. Structural analysis revealed that both dermatoxin-PD1 and T1 adopted amphipathic α-helical conformations under membrane-mimicking conditions. Functional assays demonstrated that bacterial killing was associated with membrane permeabilisation and depolarisation, with additional evidence supporting interactions with lipopolysaccharide (LPS). Notably, T1 exhibited remarkably reduced haemolytic and cytotoxic effects compared with the parent peptide, resulting in an improved selectivity profile. These findings provide additional insight into the structure–activity relationship of dermatoxin-like peptides and suggest that protease cleavage-guided truncation may represent a useful strategy for developing shorter and safer antimicrobial peptides.

## 1. Introduction

Antimicrobial resistance (AMR) has emerged as one of the most pressing global health challenges, severely threatening the effectiveness of conventional antibiotics. Among resistant bacteria, the ESKAPE pathogens (*Enterococcus faecium*, *Staphylococcus aureus*, *Klebsiella pneumoniae*, *Acinetobacter baumannii*, *Pseudomonas aeruginosa*, and *Enterobacter* species) represent a major clinical concern owing to their high virulence, multidrug resistance, and capacity for biofilm formation [1,2]. These pathogens process diverse resistance mechanisms, including enzymatic antibiotic inactivation, target modification, reduced membrane permeability, active efflux systems, and biofilm-associated persistence [3]. It has been estimated that, if current trends continue, AMR-related infections could result in up to 10 million deaths annually by 2050 [4]. Despite the urgent need for new therapeutic options, the development of novel antibiotics has remained limited, highlighting the necessity of exploring alternative classes of antimicrobial agents [5].

Antimicrobial peptides (AMPs) are cationic and amphipathic molecules that constitute essential components of innate immunity across diverse species [6,7]. Amphibians, in particular, rely heavily on skin-secreted AMPs to survive in microbe-rich environments, as their adaptive immune system is relatively underdeveloped compared with that of mammals [8,9,10]. These peptides typically exhibit broad-spectrum antimicrobial activity, rapid bactericidal kinetics, and a relatively low propensity for inducing resistance, rendering them attractive candidates for therapeutic development [11]. Several frog-derived AMPs, including dermaseptins and magainins, have been extensively investigated, with some progressing to clinical evaluation [12,13]. However, their clinical translation has often been hampered by limitations such as haemolytic activity, cytotoxicity toward mammalian cells, and insufficient stability against proteolytic degradation, resulting in a narrow therapeutic window.

Dermatoxin-PD1 (SLGSFLKGVGKGLATVGKIVADQFGKLLEAGK-NH_2_), a novel dermatoxin-like antimicrobial peptide, was identified from the skin secretions of the Mexican giant tree frog (*Pachymedusa dacnicolor*) using a molecular cloning approach. *Pachymedusa dacnicolor* is a nocturnal and arboreal phyllomedusid frog native to Mexico, whose skin secretion serves as an important component of its chemical defence against predators and environmental microorganisms [14,15]. Previous studies have identified several pharmacologically active peptide families from this species, including dermaseptins, and a recent study characterised DRS-DA2 as an antimicrobial and immunosuppressive peptide, further highlighting *P. dacnicolor* as a valuable source of peptide templates for drug discovery [16]. Based on the predicted trypsin cleavage sites within the native sequence, three cleavage-site-guided truncated analogues (T1–T3) were designed to investigate the influence of peptide truncation on antimicrobial activity and selectivity. The parent peptide and its truncated derivatives were systematically evaluated for antimicrobial activity against representative Gram-negative and Gram-positive bacteria, as well as for their haemolytic and cytotoxic effects on mammalian cells. Dermatoxin-PD1 and T1 generally exhibited greater activity against Gram-negative bacteria than against Gram-positive strains, whereas T2 and T3 showed markedly reduced antimicrobial potency. This activity profile, together with the membrane permeabilisation, depolarisation, and LPS-binding assays, supports a membrane-associated antibacterial mechanism involving interactions with lipopolysaccharide (LPS). Interactions with LPS may contribute to the preferential activity observed against some Gram-negative bacteria; however, their precise contribution to antimicrobial selectivity and reduced mammalian-cell toxicity remains to be established. Collectively, these findings show that removal of the C-terminal region can improve the selectivity of dermatoxin-PD1 while largely preserving antimicrobial activity, whereas further truncation is associated with a substantial loss of potency.

## 2. Results

### 2.1. ‘Shotgun’ Cloning and Sequence Determination of Dermatoxin-PD1 Precursor-Encoding cDNA from the Skin Secretion of Pachymedusa Dacnicolor

**Figure 1:**
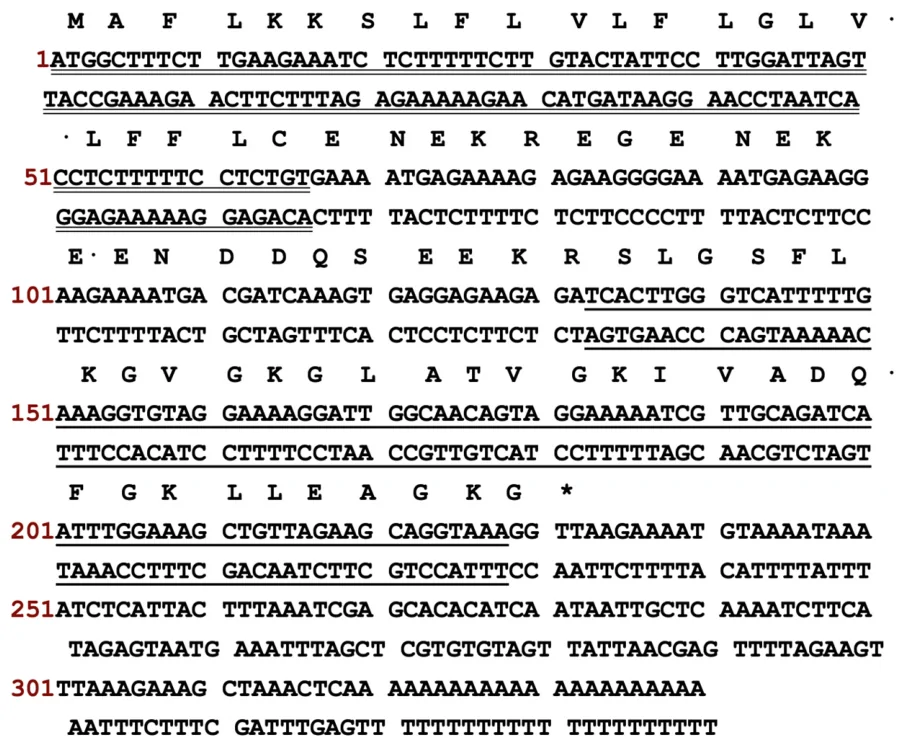
Cloned cDNA encoding the nucleotide and translated open-reading frame amino acid sequences of the biosynthetic precursor of dermatoxin-PD1. The assumed signal peptide domain is double-underlined, the mature peptide domain is single-underlined, and the termination codon is indicated by an asterisk (*).

One novel peptide precursor-encoding cDNA was consistently cloned from the *Pachymedusa dacnicolor* skin secretion-derived cDNA using the ‘shotgun’ cloning strategy. The novel gene-encoded peptide was designed as dermatoxin-PD1. As shown in Figure 1, the peptide precursor open reading frame is made up of 76 amino acid residues that have been split into five distinct domains, comprising a putative signal peptide area with 22 amino acid residues that is highly conserved, an acid peptide region with 24 amino acid residues that contains 9 glutamic acid and aspartic acid residues, a typical -Lys-Arg- (-KR-) pro-peptide convertase site and a mature peptide region with 32 amino acid residues. Also, a G residue at the C-terminus has an amide donor function that helps determine the Q residue of the mature peptide and triggers its post-translational amide modification.

**Figure 2:**
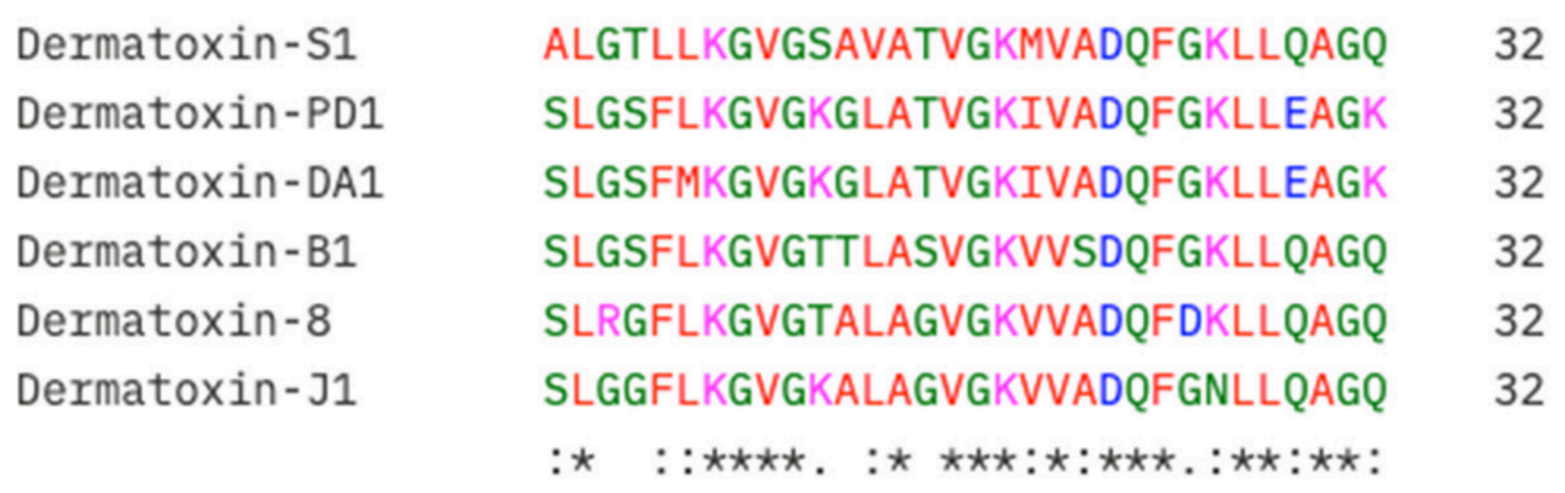
Multiple-sequence alignment of dermatoxin-PD1 with previously reported dermatoxin-related peptides. The species origin, database accession number, and literature reference for each peptide are provided where available. Asterisks (*) indicate identical residues, colons (:) indicate strongly conserved substitutions, and periods (.) indicate weakly conserved substitutions.

BLAST analysis showed that the mature dermatoxin-PD1 sequence shared high sequence similarity with previously reported dermatoxin-related peptides (Figure 2). The species origin, database accession number, and literature reference of each peptide included in the alignment are provided in Table S1. The presence of a conserved precursor organisation, a typical propeptide convertase processing site, and a C-terminal amidation signal supports the classification of dermatoxin-PD1 as a member of the dermatoxin family. It is concluded that the identified peptide is thus a member of the dermatoxin family. The cDNA sequence encoding the dermatoxin-PD1 precursor has been deposited in the GenBank database under the accession number PZ306464.

### 2.2. Peptide Design and Property Analysis

**Figure 3:**
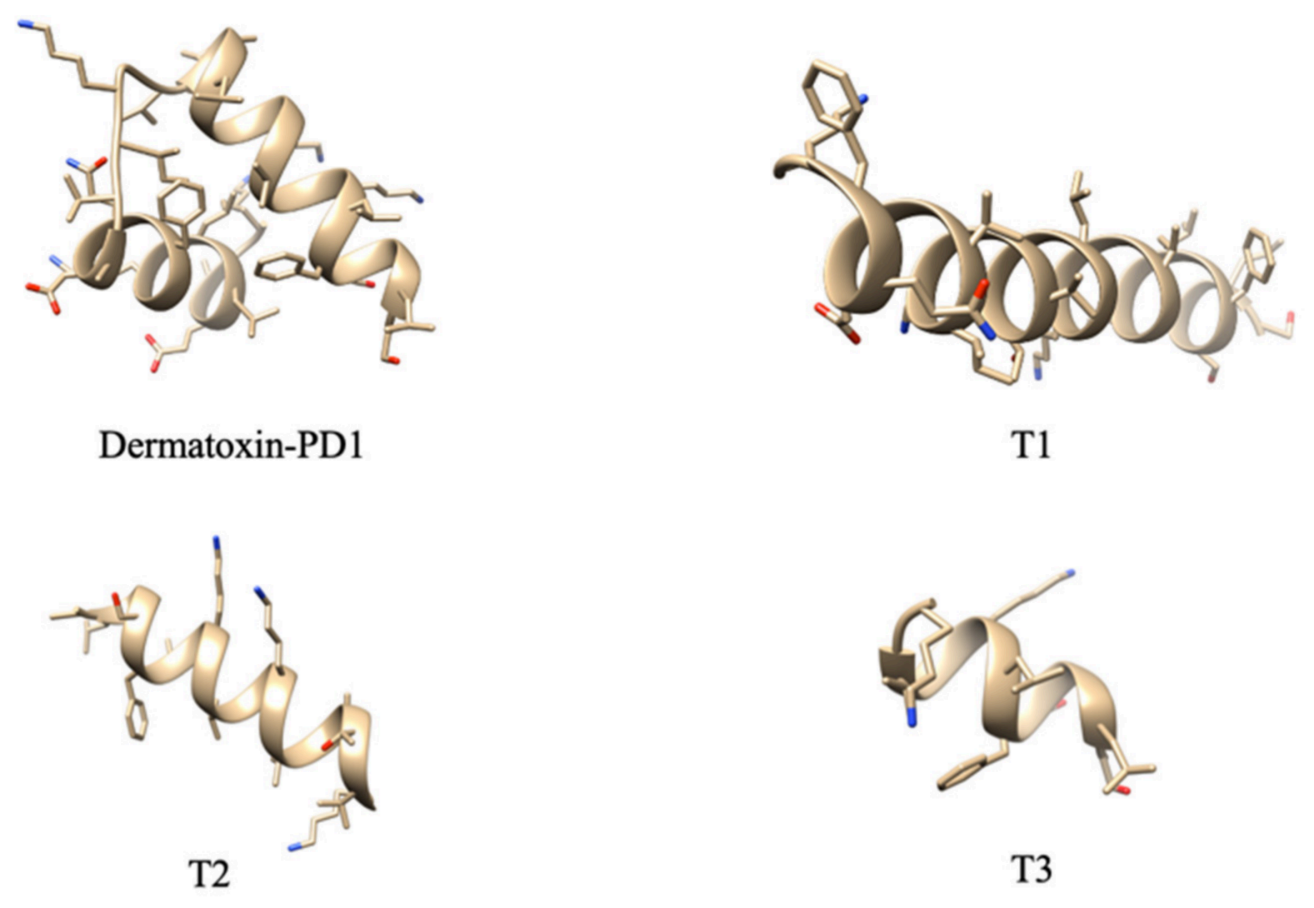
The three-dimensional structures of dermatoxin-PD1 and its analogues were predicted by PEP-FOLD4.

Because trypsin cleaves peptide bonds C-terminal to Lys/Arg residues, a trypsin-mimicking truncation workflow was used to investigate the relationship between cleavage-site-guided truncation and biological activity. Dermatoxin-PD1 was cleaved at trypsin sensitive sites to obtain the peptide analogues, T1, T2 and T3 (Table 1). After the prediction of the mature peptide sequence, the bioinformatics tool, PEP-FOLD4, was used to predict the helical structure of dermatoxin-PD1 and its truncated analogues (Figure 3). The result indicated that dermatoxin-PD1 had two regions of α-helix from Leu2 to Thr15 and from Ala21 to Ala30.

### 2.3. Secondary Structure Analysis of Dermatoxin-PD1 and T1

**Figure 4:**
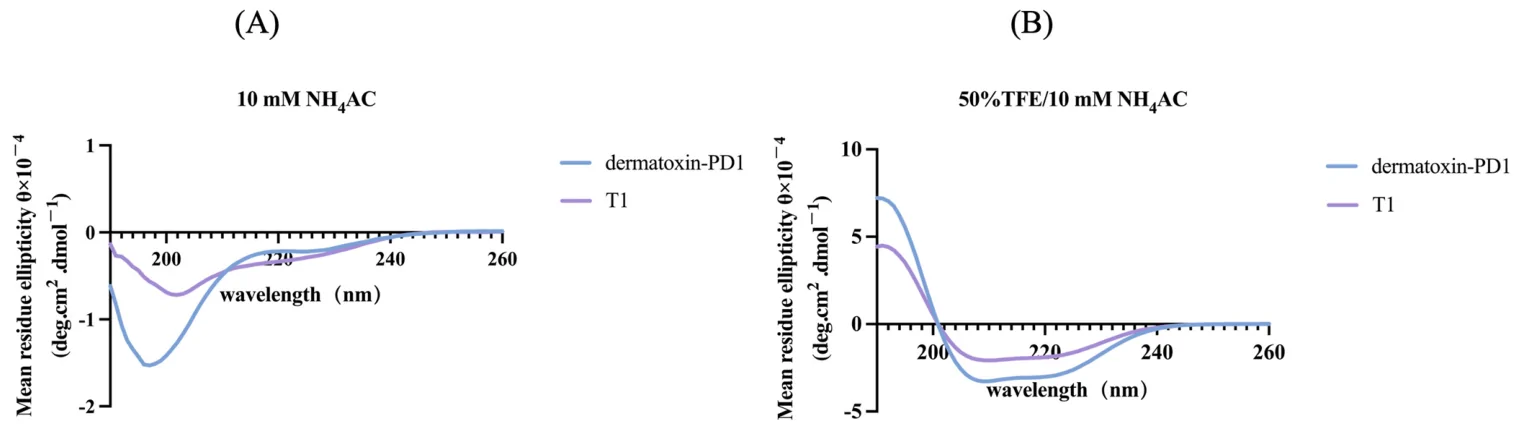
The CD spectra of dermatoxin-PD1 and T1 in (**A**) 10 mM NH_4_Ac buffer and (**B**) 50%TFE, respectively, at a concentration of 100 μM.

CD spectra were recorded to evaluate the secondary structures of dermatoxin-PD1 and the truncated analogue T1 in aqueous buffer and in the presence of 50% TFE. In 10 mM NH_4_Ac, both peptides exhibited weak CD signals without characteristic α-helical signatures, indicating predominantly unordered conformations (Figure 4A). In contrast, in 50% TFE, an organic solvent commonly used to mimic a membrane-like hydrophobic environment, both dermatoxin-PD1 and T1 displayed typical α-helical features, with negative bands at approximately 208 and 222 nm and a positive band near 190 nm (Figure 4B). Compared with dermatoxin-PD1, T1 showed slightly reduced ellipticity at 208 and 222 nm, suggesting a modest decrease in α-helical content following truncation. These CD data indicate an increased overall α-helical propensity under hydrophobic conditions; however, CD spectroscopy does not distinguish whether the induced helical structure is continuous or composed of discrete helical segments. Therefore, the CD results support TFE-induced α-helical formation at the ensemble level and are complementary to the segmented helical regions predicted by PEP-FOLD4.

### 2.4. Antimicrobial Activity of Dermatoxin-PD1 and Its Truncated Analogues

Dermatoxin-PD1 displayed potent antibacterial activity, particularly against Gram-negative bacteria, with low micromolar MIC values (2–4 µM) against multiple *E. coli* strains and *K. pneumoniae* (Table 2). Moderate activity was observed against A. baumannii, while reduced efficacy was noted against *P. aeruginosa*. The truncated analogue T1 largely retained antibacterial potency, exhibiting MIC values comparable to the parent peptide against both Gram-negative and Gram-positive bacteria. In contrast, further truncation resulted in a pronounced loss of activity, with T2 showing markedly increased MIC values and T3 being largely inactive (MIC > 128 µM) across all tested strains. For dermatoxin-PD1 and T1, MBC values were generally identical to or within one dilution of the corresponding MICs, indicating bactericidal activity. Both peptides also exhibited moderate antifungal activity against *Candida albicans*, whereas T2 and T3 were ineffective.

### 2.5. Antibiofilm Evaluation of Dermatoxin-PD1 and Its Fragments

Dermatoxin-PD1 inhibited biofilm formation of *E. coli* strains with MBIC values of 32 μM and eradicated preformed biofilms at 64 μM (MBEC) across all tested strains (Table 3). In contrast, no biofilm eradication was achieved against *S. aureus*, with MBEC values exceeding 128 μM. The truncated analogue T1 showed variable effects depending on the strain. Lower MBIC values (16 μM) were observed against *E. coli* ATCC 8739 and ATCC 13846, whereas higher concentrations were required for biofilm eradication (MBEC ≥ 64 μM or >128 μM). For *S. aureus*, both MBIC and MBEC values were 64 μM, indicating reduced efficacy compared to E. coli. In contrast, T2 and T3 showed no detectable effects on biofilm formation or eradication for either *E. coli* or *S. aureus*, with MBIC and MBEC values exceeding 128 μM.

### 2.6. Time-Killing Kinetic Studies on Dermatoxin-PD1 and Its Fragments

**Figure 5:**
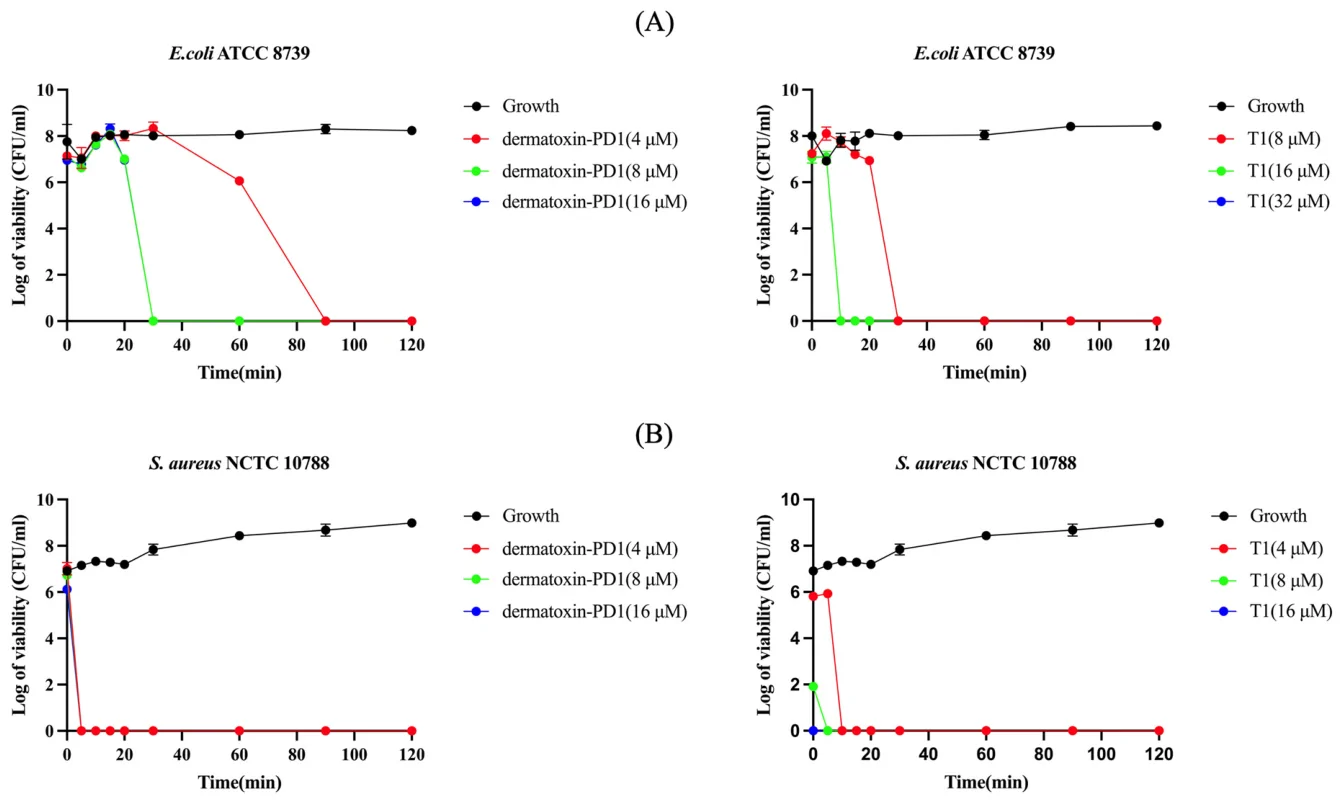
Time-killing kinetic curves of fragments dermatoxin-PD1 and T1 (1× MIC, 2× MIC and 4× MIC) on (**A**) *E. coli* (ATCC 8739) and (**B**) *S. aureus* (ATCC 10788). The standard error of the mean (SEM) is shown by the error bars in the graphs.

Time-kill assays were performed to evaluate the bactericidal kinetics of the peptides against *E. coli* ATCC 8739 and *S. aureus* NCTC 10788 and to complement the MIC data (Figure 5). Against *E. coli*, both peptides exhibited concentration-dependent killing, with substantial reductions observed within 20 min at higher concentrations. Dermatoxin-PD1 and T1 achieved complete eradication at 1×–4× MIC within 120 min, with more rapid killing observed at elevated concentrations. Against *S. aureus*, killing was generally more rapid at higher concentrations. Dermatoxin-PD1 induced an initial rapid decrease at 4 μM followed by a plateau, whereas 8–16 μM resulted in near-complete killing within ~20 min. T1 displayed rapid and sustained bactericidal activity at 8–16 μM without detectable regrowth. The effects of dermatoxin-PD1 and its analogues on bacterial viability are presented in Figure S9.

### 2.7. Condition Sensitivity Evaluation

**Figure 6:**
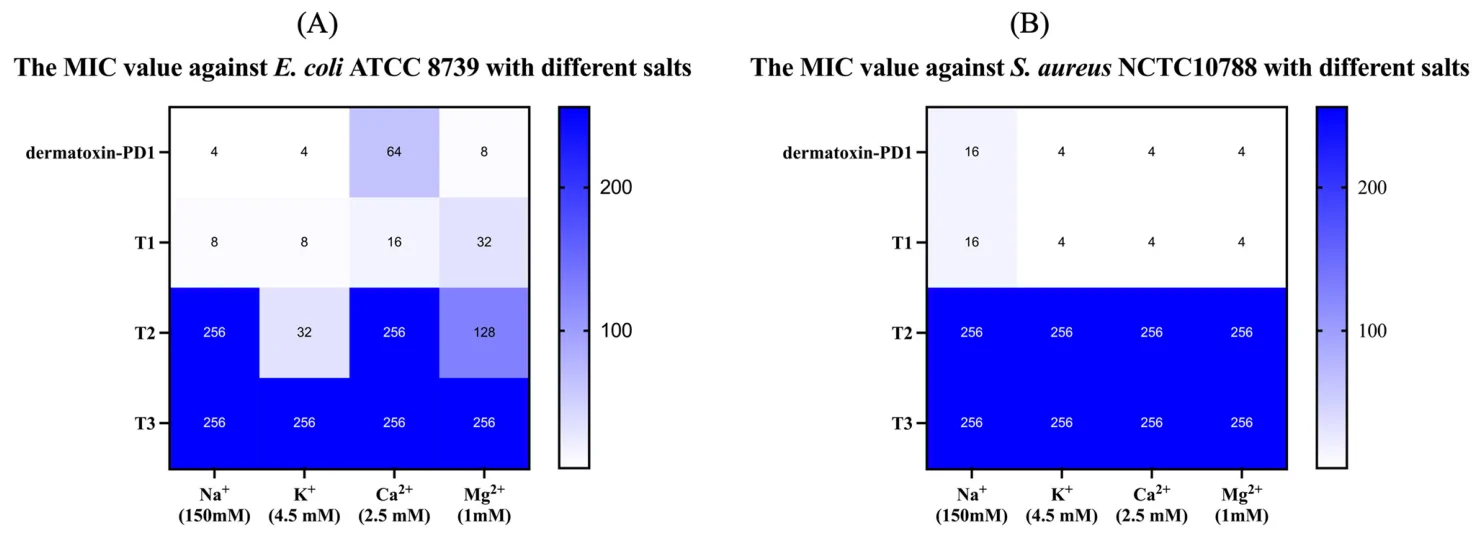
Effects of physiological salt conditions on the antimicrobial activity of dermatoxin-PD1 and its analogues against (**A**) *E. coli* ATCC 8739 and (**B**) *S. aureus* NCTC 10788. MIC/MBC values are shown in μM. When no detectable antimicrobial activity was observed >128 μM, a value of 256 μM was used for visualisation.

The salt sensitivity of Dermatoxin-PD1 and its analogues was evaluated based on MIC value under physiologically relevant ionic conditions (Figure 6). Against *S. aureus* NCTC 10788, Dermatoxin-PD1 and T1 retained low micromolar activity in the presence of K^+^, Ca^2+^, and Mg^2+^, whereas a modest reduction in activity was observed in the presence of Na^+^. In contrast, a more pronounced salt-dependent effect was observed against *E. coli* ATCC 8739. In particular, Ca^2+^ markedly reduced the activity of dermatoxin-PD1, while Mg^2+^ reduced the activity of T1. These findings suggest that divalent cations can partially attenuate the antimicrobial activity of the active peptides against Gram-negative bacteria, possibly by screening electrostatic interactions between the cationic peptides and negatively charged bacterial surface components. T2 and T3 exhibited markedly reduced activity against both strains under all salt conditions tested.

### 2.8. Membrane Mechanistic Study of Dermatoxin-PD1 and Its Fragments

**Figure 7:**
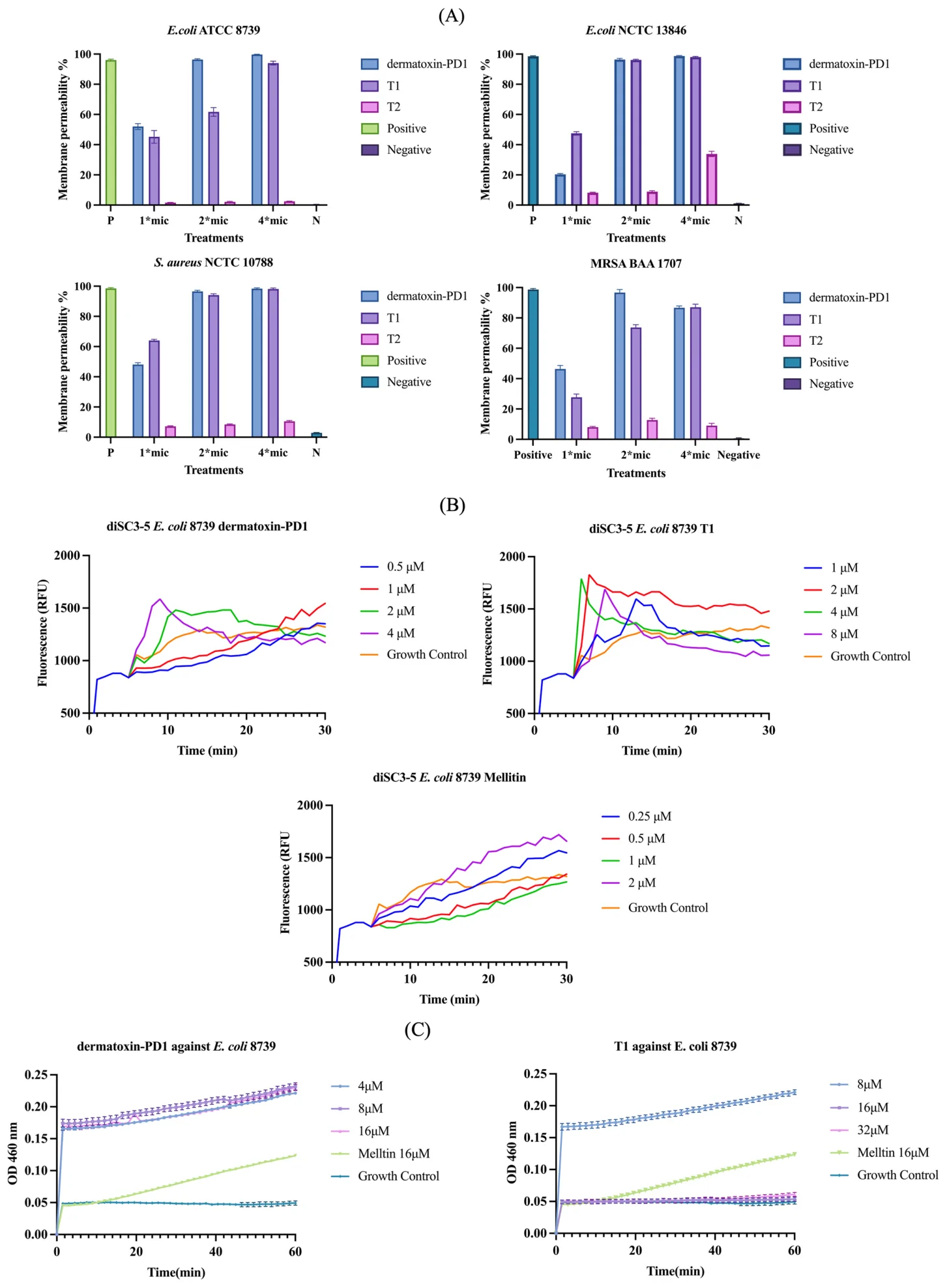
Effects of dermatoxin-PD1 and its analogues on bacterial membrane integrity and permeability. (**A**) Outer membrane permeabilization of *E. coli* ATCC 8739 and membrane permeability of *S. aureus* NCTC 10788, MRSA BAA-1707, and *E. coli* NCTC 13846, assessed by SYTOX Green uptake following treatment with dermatoxin-PD1, T1, and T2 at 1×, 2×, and 4× MIC. Melittin and untreated bacteria were used as positive and negative controls, respectively. (**B**) Membrane depolarisation of *E. coli* ATCC 8739 induced by dermatoxin-PD1, T1, and melittin at the indicated concentrations, monitored over time using the DiSC_3_-5 fluorescence assay. Increased fluorescence intensity reflects dissipation of the membrane potential. (**C**) Inner membrane permeabilisation of *E. coli* ATCC 8739 evaluated by the ONPG hydrolysis assay following treatment with dermatoxin-PD1, T1, and melittin at the indicated concentrations.

Membrane permeability induced by dermatoxin-PD1 and its analogues was evaluated using the SYTOX Green uptake assay against *E. coli* ATCC 8739, *S. aureus* NCTC 10788, MRSA BAA1707, and *E. coli* NCTC 13846 (Figure 7A). In all tested strains, dermatoxin-PD1 and T1 caused a concentration-dependent increase in membrane permeability, whereas T2 consistently exhibited minimal effects even at higher multiples of the MIC. Against *E. coli* ATCC 8739 and *S. aureus* NCTC 10788, both dermatoxin-PD1 and T1 reached high permeability levels at 2× and 4× MIC, approaching those of the positive control. Similar trends were observed for MRSA BAA1707 and *E. coli* NCTC 13846, although T1 generally produced higher permeability than dermatoxin-PD1 at equivalent concentrations.

The membrane-potential-sensitive dye DiSC_3_-5 was used to assess the depolarising effects of dermatoxin-PD1 and T1 on *E. coli* ATCC 8739 (Figure 7B). Dermatoxin-PD1 induced a moderate increase in fluorescence over time, indicating partial dissipation of membrane potential. In contrast, T1 caused a rapid and pronounced increase in fluorescence within the first few minutes following peptide addition, with a clearer concentration-dependent response. The positive control melittin produced a sustained concentration-dependent fluorescence increase, confirming effective membrane depolarisation under the assay conditions.

Inner membrane permeability was assessed using the ONPG hydrolysis assay in *E. coli* ATCC 8739 (Figure 7C). Dermatoxin-PD1 induced a concentration-dependent increase in OD_460_ at 4–16 μM over the 60 min monitoring period, indicating detectable inner-membrane permeabilisation compared with the growth control. In contrast, T1 produced a weaker ONPG response, with a transient increase observed at 8 μM, whereas OD_460_ values at 16 and 32 μM remained close to baseline. These results indicate that dermatoxin-PD1 produced a clearer ONPG hydrolysis response than T1 under the tested conditions. The positive control melittin caused a clear time-dependent increase in OD_460_, confirming effective inner-membrane permeabilisation and validating the assay.

### 2.9. LPS Binding Assay

**Figure 8:**
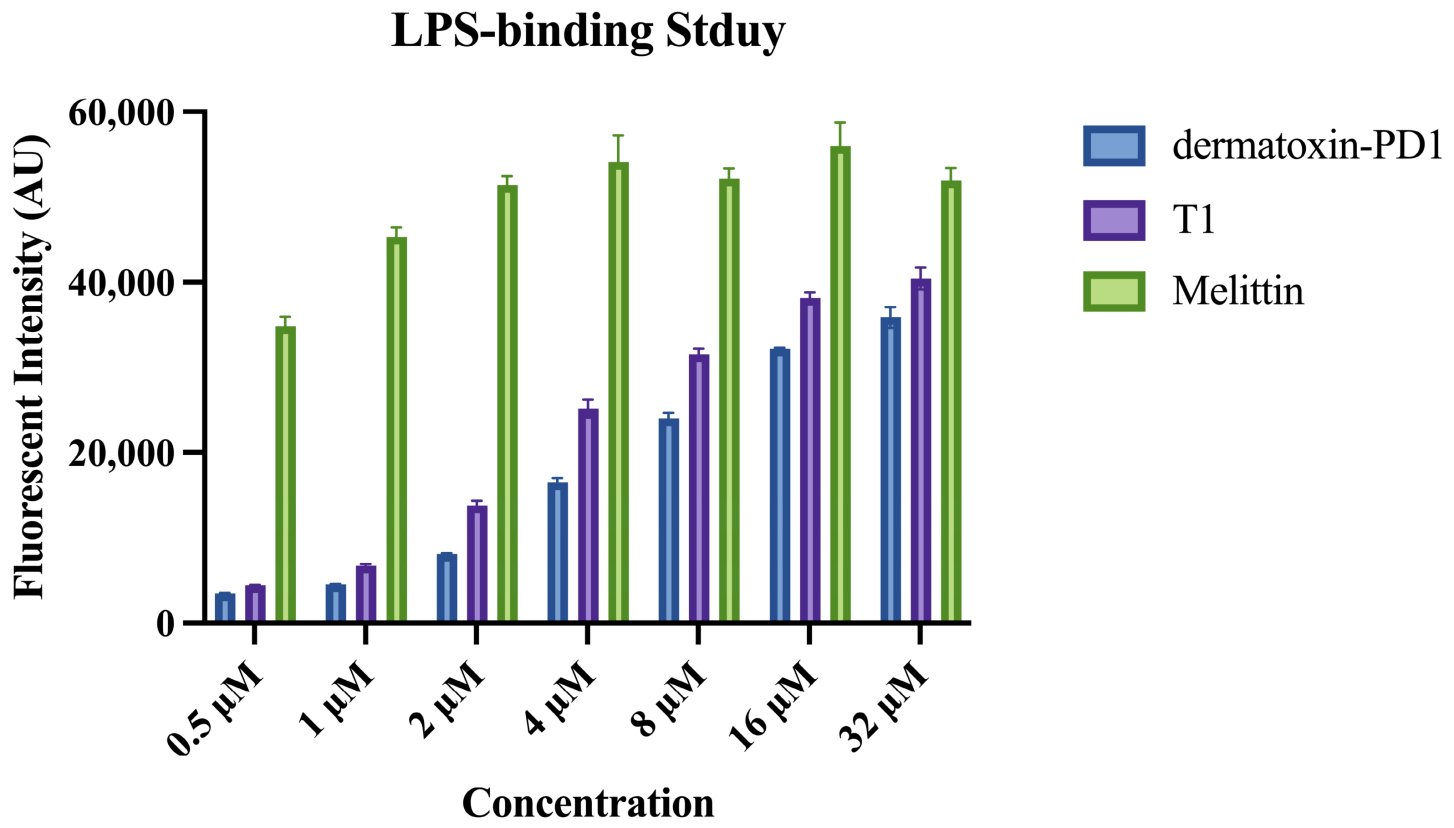
LPS-binding activity of dermatoxin-PD1 and T1 evaluated using a fluorescence-based binding assay. Peptides were tested at concentrations ranging from 0.5 to 32 μM, with melittin used as a positive control. Fluorescence intensity is expressed in arbitrary units (AU). Data are presented as mean ± SEM from independent experiments.

The LPS-binding activity of dermatoxin-PD1 and T1 was evaluated using a fluorescence-based binding assay (Figure 8). As peptide concentration increased from 0.5 to 32 μM, fluorescence intensity increased for both peptides, indicating concentration-dependent association with LPS. Across the tested concentration range, dermatoxin-PD1 and T1 exhibited comparable LPS-binding profiles, with T1 showing slightly higher fluorescence intensity at several concentrations. Melittin produced a stronger fluorescence response than both dermatoxin-derived peptides under the same assay conditions.

### 2.10. In Silico Analysis of Dermatoxin-PD1 and T1 Interactions with Lipopolysaccharide

**Figure 9:**
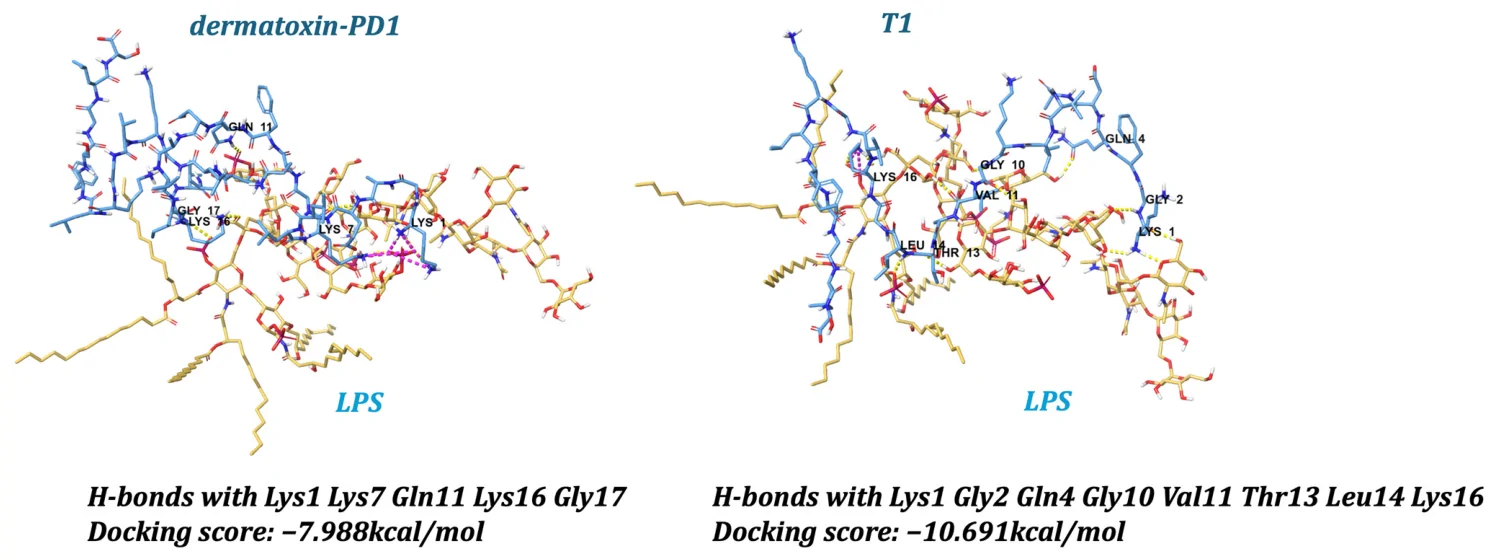
Illustrative docking poses of dermatoxin-PD1 and T1 in association with lipopolysaccharide. Peptides are shown in blue and LPS in orange. Predicted polar and hydrogen-bonding contacts are indicated by dashed lines. The models are presented solely to visualise possible peptide–LPS contact patterns and are not intended for quantitative comparison of binding affinity.

Molecular docking was performed to generate illustrative models of the possible interactions of dermatoxin-PD1 and T1 with LPS (Figure 9). Both peptides adopted predicted poses in which several polar and positively charged residues were positioned close to the LPS molecule, suggesting potential hydrogen-bonding and electrostatic contacts. In the dermatoxin-PD1 model, Lys^1^, Lys^7^, Gln^11^, Lys^16^, and Gly^17^ contributed to the predicted contact interface. In the T1 model, predicted contacts involved Lys^1^, Gly^2^, Gln^4^, Gly^10^, Val^11^, Thr^13^, Leu^14^, and Lys^16^. These docking models are presented only as qualitative illustrations of possible peptide–LPS contact patterns and should not be interpreted as quantitative estimates of binding affinity.

### 2.11. Safety Evaluation of Dermatoxin-PD1 and Its Truncated Analogues

The safety profiles of dermatoxin-PD1 and its analogues were evaluated using haemolysis, HaCaT cytotoxicity, and in vivo larval toxicity assays (Table 4 and Figure S10). dermatoxin-PD1 showed moderate haemolytic and cytotoxic effects, with an HC_50_ value of 46.47 μM and an IC_50_ value of 28.75 μM in HaCaT cells. In comparison, T1 showed lower haemolytic and cytotoxic effects, with HC_50_ and IC_50_ values of 481.1 μM and 91.34 μM, respectively. Based on the calculated selectivity indices, T1 showed higher selectivity than dermatoxin-PD1, particularly against Gram-negative bacteria. T2 and T3 showed limited haemolytic activity within the tested concentration range; however, their reduced antimicrobial activities resulted in low or undetermined selectivity indices.

The survival curves for dermatoxin-PD1 and T1 at 1×, 2×, and 4× MIC-equivalent doses remained at 100% throughout the observation period. Consequently, the different coloured treatment curves were completely superimposed and appeared as a single line in the graph. These results indicate that none of the tested concentrations of either peptide caused detectable mortality in Galleria mellonella (Figure S10).

## 3. Discussion

The development of antimicrobial peptides (AMPs) as alternatives or complements to conventional antibiotics has attracted increasing attention because many AMPs act rapidly through membrane-associated mechanisms and may exhibit a relatively low propensity for inducing resistance [15,17]. However, the clinical translation of AMPs remains constrained by multiple challenges, including high production costs, limited selectivity, susceptibility to proteolytic degradation, and inefficient modification strategies [18]. To address these limitations, shorter peptides with potent antibacterial activity have been increasingly favoured [19]. In previous studies, the truncation and modification of AMPs were often carried out by randomly selecting fragments within the sequence. However, the resulting truncated peptides frequently failed to retain their original antimicrobial activities or exhibited increased toxicity toward human erythrocytes and other cell lines [20]. In this context, rational truncation has emerged as an effective strategy to optimise peptide length while preserving biological activity.

A recurring challenge for AMP development is balancing antimicrobial potency with stability and selectivity [19,21]. Many frog-derived AMPs are enriched in Lys/Arg residues, which contribute to electrostatic attraction to bacterial membranes but may also increase susceptibility to trypsin-like proteases [22]. In the present study, dermatoxin-PD1 was identified as a dermatoxin-like peptide from *Pachymedusa dacnicolor,* and three truncated analogues were designed based on predicted Lys/Arg cleavage sites. This cleavage site-guided truncation strategy was used to evaluate how peptide length influences antimicrobial activity, membrane interaction, and safety.

The antimicrobial screening showed that dermatoxin-PD1 and the moderately truncated analogue T1 retained antibacterial activity, whereas further truncation in T2 and T3 resulted in a marked loss of function. These results suggest that the N-terminal 26 residues retained in T1 are sufficient to preserve much of the activity of the parent peptide under the tested conditions. However, because additional intermediate truncations or residue-substitution analogues were not examined, the present study does not define the minimal active pharmacophore of dermatoxin-PD1. Instead, the findings indicate that cleavage-site-guided truncation can provide a useful framework for exploring length-dependent activity in dermatoxin-like peptides. The loss of activity observed for T2 and T3 is likely related to multiple physicochemical and structural factors. Although T2 retained the same net charge as dermatoxin-PD1 and T1, its substantial reduction in length may have disrupted the amphipathic surface required for stable interaction with bacterial membranes [23,24]. T3 was even shorter and also showed a reduced net positive charge, which may further weaken its electrostatic attraction to negatively charged bacterial surfaces [25]. In addition, further truncation removed part of the predicted C-terminal helical region, which may be important for maintaining an effective membrane-active conformation. These observations suggest that peptide length, amphipathicity, cationic character, and preservation of helical propensity collectively contribute to the antimicrobial activity of dermatoxin-like peptides.

CD analysis showed that dermatoxin-PD1 and T1 adopted α-helical conformations under membrane-mimicking conditions but remained largely unordered in aqueous buffer, consistent with the behaviour of many amphipathic AMPs that undergo conformational transition upon membrane interaction [24,26]. The slightly reduced ellipticity of T1 compared with dermatoxin-PD1 suggests that truncation modestly decreased the overall α-helical content but did not abolish its ability to form an inducible helical conformation. Since CD spectroscopy reflects the overall secondary-structure propensity of the peptide population, these data should be interpreted as complementary to the segmented helical regions predicted by PEP-FOLD4 rather than as direct evidence for a fully continuous helix. Together, these findings indicate that preservation of inducible α-helical propensity is likely important for the retained activity of T1 [27].

The antimicrobial evaluation revealed that dermatoxin-PD1 exhibits potent and broad-spectrum activity, particularly against Gram-negative bacteria, with MIC values as low as 2–4 μM against multiple *E. coli* strains and *K. pneumoniae*. The truncated analogue T1 showed a similar activity profile with slightly reduced potency, whereas further truncation (T2 and T3) resulted in a marked loss of activity across most tested strains. Both dermatoxin-PD1 and T1 displayed stronger activity against Gram-negative bacteria than Gram-positive strains, with limited efficacy against *E. faecalis* and moderate activity against *S. aureus* and MRSA. In addition, both peptides exhibited moderate antifungal effects against C. albicans, albeit with lower potency than against bacteria [26]. The progressive increase in MIC and MBC values upon truncation further underscores the importance of maintaining structural integrity [28]. Collectively, these findings demonstrate a clear structure–activity relationship, in which peptide length plays a key role in determining antimicrobial potency and spectrum. The retention of activity in T1 highlights the potential of rational truncation to reduce peptide size while preserving function, whereas excessive truncation compromises efficacy [29].

Dermatoxin-PD1 and T1 exhibited moderate antibiofilm activity against *E. coli*, whereas their effects against *S. aureus* biofilms were more limited. This pattern generally followed their planktonic antibacterial profiles, in which these peptides showed relatively stronger activity against several Gram-negative strains. The reduced antibiofilm activity compared with planktonic killing also reflects the increased tolerance of biofilm-associated bacteria. In contrast, T2 and T3 showed little detectable antibiofilm activity, consistent with their weak planktonic antimicrobial effects and suggesting that sufficient peptide length and amphipathic structure are also important for antibiofilm activity.

Membrane permeabilisation is widely recognised as one of the common mechanisms of action of many antimicrobial peptides [26]. In general, cationic AMPs are initially attracted to negatively charged bacterial membranes through electrostatic interactions, followed by insertion of hydrophobic regions into the lipid bilayer, which can disrupt membrane integrity and cause membrane depolarisation [7,23]. In the present study, dermatoxin-PD1 and T1 increased SYTOX Green uptake and induced membrane depolarisation, whereas T2 showed minimal permeability effects, consistent with its reduced antimicrobial activity. These results support a membrane-associated mode of action for dermatoxin-PD1 and T1. Although the ONPG assay showed a clearer inner-membrane permeabilisation response for dermatoxin-PD1 than for T1, this result should be interpreted together with the SYTOX Green, DiSC_3_-5, MIC, and time-kill data rather than in isolation.

LPS, a major component of the outer membrane of Gram-negative bacteria, plays an important role in the initial interaction between cationic AMPs and Gram-negative bacterial cells [30]. The negative charge of LPS can facilitate peptide binding and contribute to outer-membrane permeabilisation [31]. In the present study, fluorescence-based LPS-binding assays showed that both dermatoxin-PD1 and T1 interacted with LPS in a concentration-dependent manner. Molecular docking further suggested that both peptides could form predicted peptide–LPS complexes, with T1 showing a lower predicted binding energy than dermatoxin-PD1 under the applied docking conditions. However, this stronger predicted docking score did not directly translate into lower MIC values against all Gram-negative strains. This discrepancy indicates that LPS binding is likely only one component of antibacterial activity, while other factors, including peptide conformation, membrane insertion, aggregation behaviour, and downstream membrane disruption, may also influence antimicrobial potency. Therefore, the LPS-binding and docking results should be interpreted as supportive evidence for peptide–LPS interaction rather than as direct predictors of antibacterial efficacy [31,32].

Safety evaluation showed that T1 had lower haemolytic and cytotoxic effects than dermatoxin-PD1, resulting in improved selectivity indices, particularly against Gram-negative bacteria. This finding suggests that moderate truncation can improve the balance between antimicrobial activity and host-cell safety. In the *Galleria mellonella* larval model, neither dermatoxin-PD1 nor T1 caused detectable mortality at doses up to 4× MIC over the observation period, further supporting their low acute toxicity under the tested conditions.

The improved safety profile of T1 should not be attributed solely to LPS binding [30,31]. Although LPS interaction may contribute to the activity of dermatoxin-PD1 and T1 against Gram-negative bacteria, the reduced haemolytic and cytotoxic effects of T1 may also be associated with changes in peptide length, hydrophobic surface exposure, amphipathic organisation, and interaction with mammalian cell membranes [33]. Further biophysical studies using bacterial and mammalian membrane models would be required to clarify the precise basis of this improved selectivity. Taken together, these findings suggest that cleavage-site-guided truncation can improve the balance between antimicrobial activity and host-cell safety, while preserving an inducible amphipathic structure and sufficient cationic character appears important for maintaining activity [34].

Overall, this study provides new insights into the structure–activity relationships of dermatoxin peptides and highlights rational truncation as a practical strategy for developing shorter, safer, and more selective antimicrobial agents.

## 4. Materials and Methods

### 4.1. Acquisition of the Skin Secretion from the Frog, Pachymedusa Dacnicolor

Skin secretions from adult *Pachymedusa dacnicolor* were obtained by low-voltage transcutaneous electrical stimulation (2–5 V), followed by gentle manual massage of the dorsal skin. The secretions were immediately rinsed from the skin surface using ice-cold sterile deionized water, collected into pre-chilled containers on ice, snap-frozen in liquid nitrogen, and lyophilised using a bench-top freeze dryer. The resulting dried material was stored at −20 °C until further use. All procedures were conducted in accordance with the UK Animals (Scientific Procedures) Act 1986 (Project Licence PPL2694) and were approved by the Animal Welfare and Ethical Review Body (AWERB) of Queen’s University Belfast. Electrical stimulation intensity and duration were minimized to reduce animal distress.

### 4.2. ‘Shotgun’ Cloning and Sequence Determination of Dermatoxin-PD1 Precursor-Encoding cDNA

Approximately 5 mg of lyophilised skin secretion was dissolved in 1 mL lysis/binding buffer, and poly(A) mRNA was isolated from the clarified lysate using the Dynabeads mRNA Direct™ kit (ThermoFisher, Waltham, MA, USA). First-strand cDNA was synthesized on bead-bound mRNA, and rapid amplification of cDNA ends (RACE) was performed with the SMART-RACE kit (Clontech, Oxford, UK) to obtain the prepropeptide-encoding sequence. RACE employed the kit-supplied nested universal primer (NUP) together with a degenerate forward primer designed against the conserved signal peptide of dermatoxin family precursors (5′-DBTTYDRAAHYTTTTTYKHWTYYKDDYYYYTTTYYBT-3′, Y=T/C, K=G/T, R=A/G, W=A/T, H=A/C/T, B=C/G/T, N=A/T/C/G, D=A/G/T,). PCR products were resolved by agarose gel electrophoresis, purified (Concert™ Rapid PCR Purification, Life Technologies, Carlsbad, CA, USA), ligated into pGEM-T Easy (Promega, Madison, WI, USA), and Sanger-sequenced with vector primers. The translated open reading frame (ORF) was verified by BLAST (NCBI) and aligned with related amphibian AMP precursors.

### 4.3. In Silico Analysis of Physicochemical Properties and Molecular Docking Simulation

Secondary-structure models for dermatoxin-PD1 and analogues were generated with PEP-FOLD4 (top-5 models; best sOPEP score retained). Physicochemical descriptors, including length, molecular weight, net charge, and α-helical hydrophobic moment (μH), were computed with HeliQuest and ExPASy ProtParam; helical wheel projections were obtained from HeliQuest using an α-helical window. Sequence similarity searches of the parent precursor were performed against UniProtKB using BLAST, and related amphibian AMP precursors were aligned with Clustal Omega to contextualize family membership. All web tools were run with default parameters unless stated otherwise, and versions/access dates are reported in the Supplementary Information.

Peptide–LPS docking was carried out to explore the interaction between the peptides and LPS. The three-dimensional structures of dermatoxin-PD1, T1, and the reference peptide buforin II were generated using PEP-FOLD4, and the top-ranked models were selected for subsequent analysis. The LPS structure was obtained from the Protein Data Bank. Prior to docking, all structures were prepared by removing solvent molecules and adding polar hydrogens. Docking calculations were performed using AutoDock Vina (1.1.2) with default settings, with the search space covering the entire LPS molecule. The binding pose with the lowest predicted binding energy was selected, and hydrogen bond interactions were examined and visualized using PyMOL 2.6.2.

### 4.4. Peptide Synthesis, Purification, and Mass Spectrometric Characterisation

The peptides were synthesised by solid-phase peptide synthesis (SPPS) using a Tribute^®^ automated peptide synthesiser (Protein Technologies, Tucson, AZ, USA) on Rink amide MBHA resin (Fluorochem, Hadfield, UK). Crude peptides were purified by reversed-phase high-performance liquid chromatography (RP-HPLC) using a Waters system (Milford, MA, USA) equipped with an analytical C5 column (Jupiter C5, 250 × 4.6 mm; Phenomenex, Macclesfield, Cheshire, UK). Peptides were eluted at a flow rate of 1.0 mL·min^−1^ using a linear gradient of 0–100% solvent B over 80 min, where solvent A was water containing 0.05% (*v*/*v*) trifluoroacetic acid and solvent B was acetonitrile containing 0.05% (*v*/*v*) trifluoroacetic acid. The purified peptides were characterized by MALDI-TOF mass spectrometry (4800 MALDI TOF/TOF, Applied Biosystems, Foster, CA, USA). The analysis was performed in the positive detection mode employing α-cyano-4-hydroxycinnamic acid (CHCA, Sigma-Aldrich, St. Louis, MO, USA) as the matrix.

### 4.5. Secondary Structure Analysis

Circular dichroism (CD) analyses were performed using a JASCO J815 Spectropolarimeter (JASCO Inc., Great Dunmow, UK) with a 1 mm path-length quartz cuvette. Each sample was analysed at 20 °C over the wavelength range of 190–260 nm. The instrument parameters were set to a scanning speed of 100 nm/min, a bandwidth of 1 nm, and a data pitch of 0.5 nm, with measurements taken as the average of three accumulations. Peptide samples were prepared at a final concentration of 100 μM in 10 mM ammonium acetate buffer and 50% TFE in 10 mM ammonium acetate buffer.

### 4.6. Determination of MIC and MBC Against Planktonic and Biofilm-Forming Bacteria

Antimicrobial activity was evaluated against Gram-positive bacteria *Staphylococcus aureus* (ATCC 6538), *methicillin-resistant Staphylococcus. aureus* (MRSA, NCTC 12493 and ATCC BAA 1707), and *Enterococcus faecium* (NCTC 12697); Gram-negative bacteria *Escherichia coli* (ATCC 8739,NCTC 13846 and ATCC 2340), *Klebsiella pneumoniae* (ATCC 43816), *Pseudomonas aeruginosa* (ATCC CRM 9027), and *Acinetobacter baumannii* (ATCC BAA-747); and the fungus *Candida albicans* (ATCC 10231). Peptides were dissolved in DMSO to 12,800 µM and two-fold serially diluted (12,800–100 µM). Log-phase cultures were prepared by incubating overnight cultures in appropriate media at 37 °C with shaking. After 20–24 h incubation, growth was assessed at OD_550_, and viability was calculated relative to the negative control. MBCs were determined by plating 10 µL from wells showing no visible growth; the lowest concentration yielding no colonies was recorded as the MBC. Biofilm susceptibility was assessed using *S. aureus* (ATCC 10788) and *E. coli* (ATCC CRM 8739, NCTC13846, ATCC 2340). Log-phase cultures (5 × 10^5^ CFU·mL^−1^) were used to form biofilms in LB (for *S. aureus*) or LB supplemented with 1% (*w*/*v*) glucose (for *E. coli*). For MBIC determination, bacterial suspensions were co-incubated with peptides (100–51,200 µM; DMSO ≤ 1% *v*/*v*) for 24 h at 37 °C, 200 rpm. For MBEC, biofilms were pre-established for 24 h prior to peptide exposure. Biofilm biomass was quantified by crystal violet staining (A595 with MBIC defined as inhibition of biofilm formation and MBEC as reduction of established biofilms to background levels. All assays were performed in triplicate in at least three independent experiments.

### 4.7. Time-Killing Kinetics Assay

Time-killing assays were performed with *E. coli* ATCC 8739 and *S. aureus* NCTC 10788. Bacterial suspensions were prepared as described for the antimicrobial assays (Nutrient Broth (NB; Sigma-Aldrich, St. Louis, MO, USA) for *E. coli*; tryptic soy broth (TSB; Sigma-Aldrich, St. Louis, MO, USA) for *S. aureus*). Cultures were exposed to peptides at 1×, 2×, and 4× MIC. At 0, 5, 10, 15, 20, 30, 60, 90, and 120 min, aliquots were serially diluted in PBS (10^−1^–10^−4^) and 10 µL of each dilution was plated on Nutrient agar (*E. coli*) or Tryptic Soy Agar (TSA) (*S. aureus*). Each dilution was plated in triplicate, and the experiment was repeated three times. Colony counts were used to determine CFU mL^−1^.

### 4.8. Condition Sensitivity Assay

The antimicrobial activity of peptides under saline and serum conditions was evaluated using the broth microdilution method as described for MIC determination. For salt sensitivity assays, the test medium was supplemented with NaCl (150 mM), KCl (4.5 mM), MgCl_2_ (1 mM), and CaCl_2_ (2 mM), either individually or in combination as specified. For serum sensitivity assays, the medium was supplemented with 10% (*v*/*v*) fetal bovine serum (FBS; Gibco, Waltham, MA, USA). Peptide stock solutions and vehicle controls were prepared according to the MIC protocol (final DMSO concentration ≤ 1%, *v*/*v*). Incubation conditions and MIC endpoints were identical to those used in the standard antimicrobial assays. All experiments were performed in triplicate and repeated in at least three independent experiments.

### 4.9. Membrane Interaction Assays

#### 4.9.1. SYTOX Green Permeability Assay

Membrane permeability assays were performed using *E. coli* ATCC 8739, *E. coli* NCTC 13846, *S. aureus* NCTC 10788, and MRSA ATCC BAA 1707. Overnight cultures grown in TSB were subcultured for 2.5 h, harvested, washed, and resuspended in 5% TSB/0.85% NaCl to ~1 × 10^8^ CFU mL^−1^. Bacterial suspensions were incubated with peptides (based on MIC values) in black 96-well plates for 2 h at 37 °C. SYTOX™ Green (5 µM, Thermo Fisher Scientific, Waltham, MA, USA) was added and incubated for 5 min in the dark, with melittin (8 µM) as a positive control. Fluorescence was measured at 485/528 nm using a Synergy HT microplate reader (BioTek, Winooski, VT, USA).

#### 4.9.2. Dynamic Inner Membrane Permeability Assay (ONPG)

ONPG permeability assays were conducted using *E. coli* ATCC CRM 8739. Overnight cultures grown in Luria–Bertani medium (LB; Sigma-Aldrich, St. Louis, MO, USA) supplemented with 2% (*w*/*v*) lactose were subcultured for 2 h, harvested, and resuspended in pre-warmed PBS containing 1.5 mM ONPG (Thermo Fisher Scientific, Waltham, MA, USA) (OD_600_ = 0.5, diluted 1:10). Bacterial suspensions were incubated with peptides (1–4× MIC) in clear 96-well plates, with melittin (16 µM) and PBS as positive and negative controls, respectively. Absorbance at 460 nm was monitored every 1.5 min for 60 min following equilibration at 37 °C.

#### 4.9.3. Cytoplasmic Membrane Depolarisation (DiSC_3_-5)

Cytoplasmic membrane depolarisation was assessed using the membrane potential–sensitive dye DiSC_3_-5 (Thermo Fisher Scientific, Waltham, MA, USA). *E. coli* ATCC CRM 8739 was grown overnight in LB and subcultured for 2 h to mid-log phase. Cells were harvested (2000× *g*, 10 min, 4 °C), washed three times with 5 mM HEPES buffer containing 20 mM glucose (pH 7.2–7.4), and resuspended in 5 mM HEPES supplemented with 0.1 M KCl to an OD_600_ of 0.05. DiSC_3_-5 was added to a final concentration of 20 µM and incubated at room temperature for 30–60 min to allow dye equilibration. Aliquots of stained cells (90 µL) were mixed with peptide solutions (10 µL) in black 96-well plates, with HEPES buffer and melittin (0.25–4 µM) as negative and positive controls, respectively. Fluorescence was monitored at 485/645 nm every minute for 30 min using a microplate reader.

### 4.10. Lipopolysaccharide (LPS) Binding Assay

Peptide binding to LPS was evaluated using a BODIPY TR cadaverine (BC) displacement assay. BC (5 µg mL^−1^, Thermo Fisher Scientific, Waltham, MA, USA) was pre-incubated with LPS (50 µg mL^−1^, Tris–HCl buffer, pH 7.4) at room temperature for 4 h. Peptides at increasing concentrations were subsequently added and incubated at 37 °C for 1 h. Fluorescence was recorded at excitation/emission wavelengths of 580/620 nm. Melittin was included as a positive control, and all experiments were performed independently in triplicate.

### 4.11. Drug Safety Analysis

#### 4.11.1. Haemolysis Assay

Haemolytic activity was evaluated using fresh defibrinated horse erythrocytes. Blood was washed three times with PBS (1000× *g*, 5 min) and resuspended to 2% (*v*/*v*) in PBS. Peptides (2–256 µM) were incubated with erythrocyte suspensions for 2 h at 37 °C in the dark. PBS and 1% (*v*/*v*) Triton X-100 were used as negative (0%) and positive (100%) controls, respectively. Following centrifugation (1000× *g*, 5 min), supernatants were transferred to 96-well plates and absorbance was measured at 570 nm using a Synergy HT microplate reader. Percentage haemolysis was calculated relative to the negative and positive controls.

#### 4.11.2. Cytotoxicity Study on HaCaT

HaCaT keratinocytes (ATCC, Manassas, VA, USA) were maintained in Minimum Essential Medium (MEM) supplemented with 10% fetal bovine serum and 1% penicillin–streptomycin at 37 °C in a 5% CO_2_ atmosphere. Cells were seeded into 96-well plates (1 × 10^4^ cells per well), allowed to adhere for 24 h, and serum-starved for 4 h prior to treatment. Peptides (1 µM–100 µM) prepared in serum-free medium were added and cells were incubated for 24 h. PBS and 0.1% (*v*/*v*) Triton X-100 served as negative and positive controls, respectively. Cell viability was assessed using the MTT assay. MTT solution (5 mg mL^−1^, 10 µL per well) was added and plates were incubated for 2 h at 37 °C in the dark. Formazan crystals were solubilised in DMSO and absorbance was measured at 570 nm using a Synergy HT microplate reader. Cell viability was expressed as a percentage of the negative control, and IC_50_ values were determined by nonlinear regression. Experiments were conducted in triplicate with at least three independent repeats.

#### 4.11.3. In Vivo Toxicity Study

In vivo toxicity of dermatoxin-PD1 was evaluated using the Galleria mellonella (*G. mellonella*) larvae model as performed previously [14,35]. In short, the larvae in the 5th instar, with weights ranging from 200 to 250 mg, were selected and injected with 10 μL of peptide solutions at different concentrations (corresponding to the concentration of 1-, 2-and 4-fold MIC). Then, larvae were incubated for seven days at 37 °C. The number of surviving larvae was counted daily. Any changes in body colour were considered toxic signs. The test was repeated three times.

### 4.12. Statistical Analysis

Prism software was used for the data analysis (Version 10.0; GraphPad Software Inc., San Diego, CA, USA). The standard error of the mean (SEM) for each set of data in the three replicates from three experiments was shown by the error bars surrounding the mean data points in the graphs.

## 5. Conclusions

A novel dermatoxin-like peptide, dermatoxin-PD1, was identified and systematically optimized through rational truncation. The truncated analogue T1 retained potent antimicrobial activity, particularly against Gram-negative bacteria, while exhibiting markedly reduced cytotoxicity and improved selectivity. Mechanistic studies suggest that its activity is associated with membrane disruption and LPS interaction. These findings demonstrate that preservation of a minimal amphipathic α-helical domain is sufficient for antimicrobial function and highlight rational truncation as an effective strategy for developing shorter and safer antimicrobial peptides.

## Figures and Tables

**Table 1:** Physicochemical characteristics of dermatoxin-PD1 and its analogues.

Peptide	Sequence	Net Charge	Hydrophobicity (H)	Hydrophobic Moment <µH>
dermatoxin-PD1	SLGSFLKGVGKGLATVGKIVADQFGKLLEAGK-NH_2_	+4	0.377	0.512
T1	SLGSFLKGVGKGLATVGKIVADQFGK-NH_2_	+4	0.384	0.512
T2	SLGSFLKGVGKGLATVGK-NH_2_	+4	0.381	0.562
T3	SLGSFLKGVGK-NH_2_	+3	0.395	0.653

**Table 2:** Summary of MICs and MBCs of the AMP dermatoxin-PD1 and its analogues.

Strains	MICs/MBCs(µM)			
	Dermatoxin-PD1	T1	T2	T3
*E. coli* ATCC 8739	4/4	8/8	32/64	>128
*E. coli* NCTC 13846	2/2	2/4	16/32	>128
*E. coli* ATCC 2340	2/2	2/4	32/32	>128
*K. pneumoniae* ATCC 43816	4/4	8/32	128/>128	>128
*P. aeruginosa* ATCC CRM9027	32/32	16/64	64/64	>128
*A. baumannii* BAA 747	8/8	16/32	>128	>128
MRSA NCTC 12493	8/16	8/8	>128	>128
MRSA ATCC BAA 1707	16/16	8/8	>128	>128
*E. faecalis* NCTC 12697	>128	>128	>128	>128
*S. aureus* NCTC 10788	4/4	4/4	>128	>128
*C. albicans* ATCC 10231	8/8	16/16	128/128	>128

**Table 3:** The MBICs and MBECs of dermatoxin-PD1 and its analogues against reference bacteria.

Strains	MBICs/MBECs(µM)			
	Dermatoxin-PD1	T1	T2	T3
*E. coli* ATCC 8739	32/64	16/64	>128	>128
*E. coli* NCTC 13846	32/64	32/>128	>128	>128
*E. coli* ATCC 2340	32/64	16/>128	>128	>128
*S. aureus* NCTC 10788	32/>128	64/64	>128	>128

**Table 4:** Haemolytic activity, cytotoxicity, and selectivity index of Dermatoxin-PD1 and its truncated analogues.

Peptides	HC_50_ ^1^ (µM)	IC50 ^2^	GM ^3^	GM_MIC_ ^4^			SI ^5^		
		HaCaT	HC_50_& IC_50_	Gram−	Gram+	All	Gram−	Gram+	All
Dermatoxin-PD1	46.47	28.75	36.55	3.48	19.03	8.14	10.5	1.92	4.49
T1	481.1	91.34	209.63	5.28	16	9.19	39.7	13.1	22.81
T2	256	>128	256	55.72	256	119.43	4.59	1	2.14
T3	256	>128	-	-	-	-	-	-	-

^1^ HC_50_: 50% haemolytic concentration; ^2^ IC_50_: 50% inhibitory concentration against HaCaT cells. ^3^ GM (HC_10_ & IC_50_): geometric mean of HC_10_ and IC_50_. ^4^ GMMIC: geometric mean of MIC values. All values are in μM. ^5^ SI = GM (HC_10_ & IC_50_)/GM_MIC_.

## Data Availability

The dermatoxin-PD1 biosynthetic precursor-encoding cDNA has been deposited in the NCBI Database under the accession number: PZ306464.

## Supplementary Materials

The following supporting information can be downloaded at: https://www.mdpi.com/article/10.3390/molecules31162748/s1, Table S1: Origin and literature information of dermatoxin-related peptides included in the sequence alignment; Figure S1: Helical-wheel projections of dermatoxin-PD1 generated using HeliQuest with an α-helical window; Figure S2: Helical-wheel projections of T1 generated using HeliQuest with an α-helical window; Figure S3: Helical-wheel projections of T2 generated using HeliQuest with an α-helical window; Figure S4: Helical-wheel projections of T3 generated using HeliQuest with an α-helical window; Figure S5: Analytical characterisation of synthetic dermatoxin-PD1. (a) Analytical RP-HPLC chromatogram showing the purity of dermatoxin-PD1. (b) Mass spectrum confirming the molecular mass of the synthesised peptide; Figure S6: Analytical characterisation of synthetic T1. (a) Analytical RP-HPLC chromatogram showing the purity of T1. (b) Mass spectrum confirming the molecular mass of the synthesised peptide; Figure S7: Analytical characterisation of synthetic T2. (a) Analytical RP-HPLC chromatogram showing the purity of T2. (b) Mass spectrum confirming the molecular mass of the synthesised peptide; Figure S8: Analytical characterisation of synthetic T3. (a) Analytical RP-HPLC chromatogram showing the purity of T3. (b) Mass spectrum confirming the molecular mass of the synthesised peptide; Figure S9: Changes in bacterial viability following peptide treatment, expressed as log_10_(CFU/mL) differences between 0 min and 120 min. Time-killing effects were evaluated against (A) E. coli ATCC 8739 and (B) S. aureus NCTC 10788 at the indicated peptide concentrations; Figure S10: Survival of Galleria mellonella larvae following treatment with dermatoxin-PD1 and T1 at 1×, 2×, and 4× MIC-equivalent doses. Refs. [36,37,38] are cited in the Supplementary Materials.
