# Supplementary material for: Identification and Structure–Activity Relationship of a Novel Dermatoxin-like Antimicrobial Peptide with Partial LPS-Mediated Membrane Interaction
Source: Molecules. 2026 Aug 7;31(16):2748. doi: 10.3390/molecules31162748 (PMC13515218; doi:10.3390/molecules31162748)
Supplement: Supplementary file 1 [file molecules-31-02748-s001.zip › molecules-4447401-supplementary.pdf]

Table S1. Origin and literature information of dermatoxin-related peptides included in the sequence alignment.

Peptide	Species origin	UniProt / database accession	Reference
Dermatoxin-DA1	*Agalychnis dacnicolor*	UniProt: O93451	[1]
Dermatoxin-B1	*Phyllomedusa bicolor*	UniProt: Q9PT75	[2]
Dermatoxin-8	*Phyllomedusa tarsius*	UniProt: P84928.1	Direct submission to UniProtKB, 2006
Dermatoxin-J1	*Phasmahyla jandaia*	UniProt: P86621	[3]
Dermatoxin-S1	*Phyllomedusa sauvagii*	UniProt: Q5DVA5	[4]

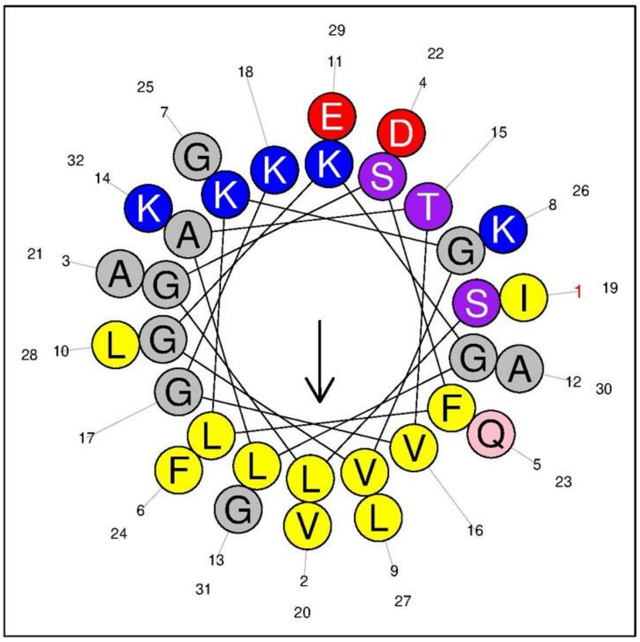

Figure S1. Helical-wheel projections of dermatoxin-PD1 generated using HeliQuest with an α-helical window. Residues are coloured according to physicochemical class: hydrophobic residues, yellow; positively charged residues, blue; negatively charged residues, red; polar uncharged residues, purple; and glycine residues, grey. Arrows indicate the direction of the calculated hydrophobic moment (μH).

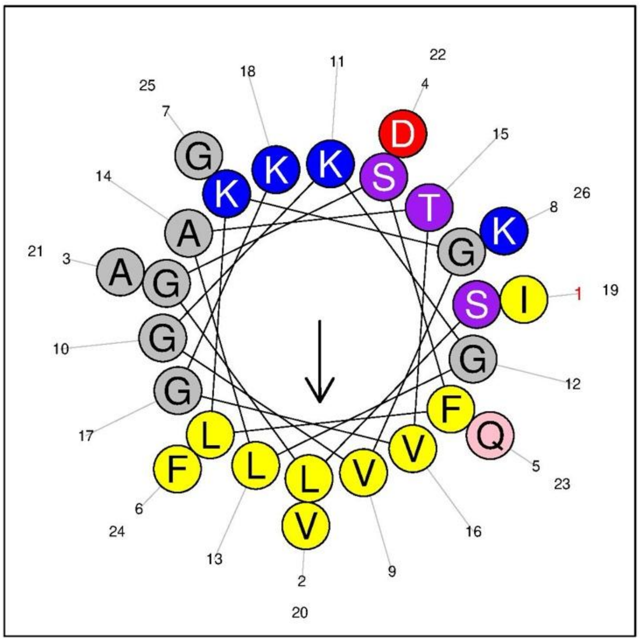

Figure S2. Helical-wheel projections of T1 generated using HeliQuest with an α-helical window.

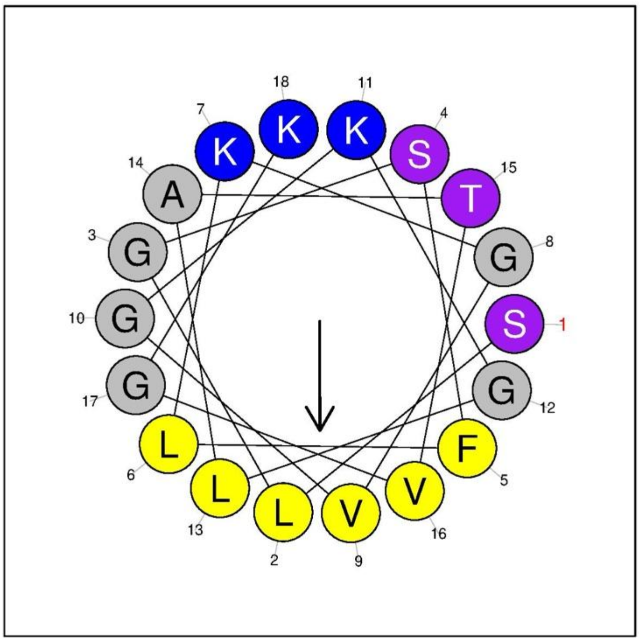

Figure S3. Helical-wheel projections of T2 generated using HeliQuest with an α-helical window.

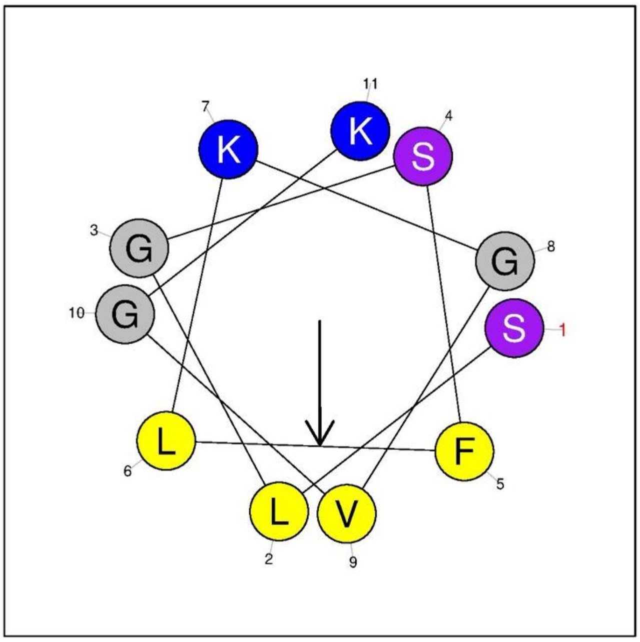

Figure S4. Helical-wheel projections of T3 generated using HeliQuest with an α-helical window.

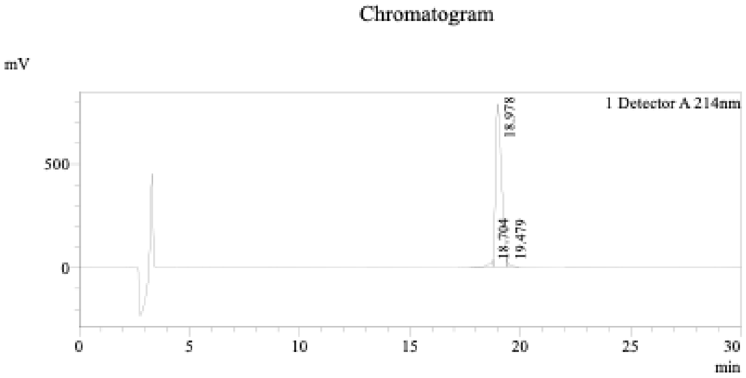

(a)

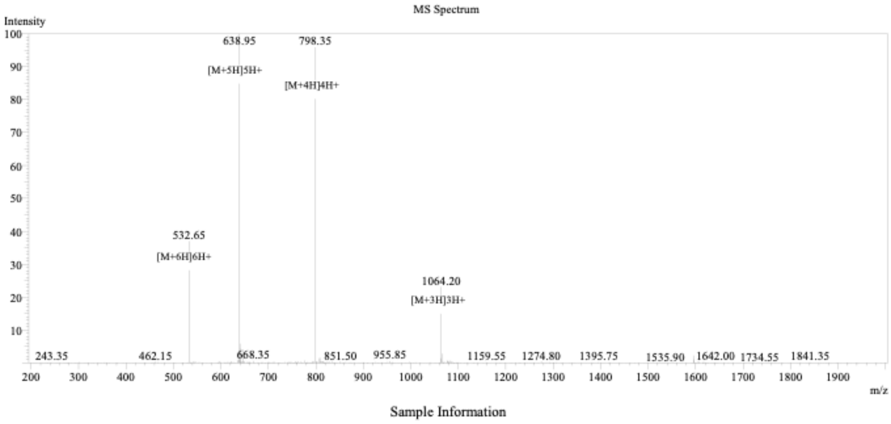

(b)

Figure S5. Analytical characterisation of synthetic dermatoxin-PD1. (a) Analytical RP-HPLC chromatogram showing the purity of dermatoxin-PD1. (b) Mass spectrum confirming the molecular mass of the synthesised peptide.

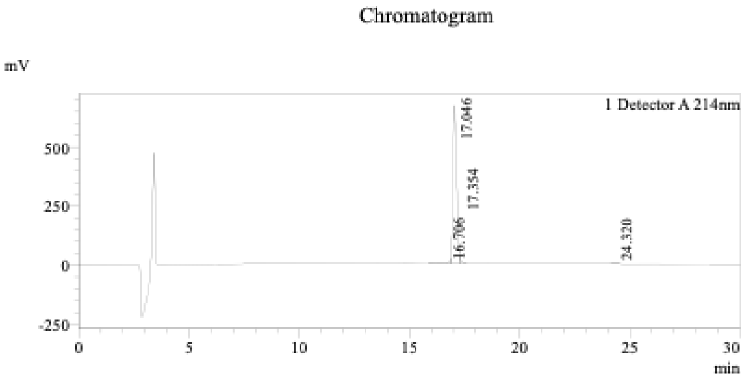

(a)

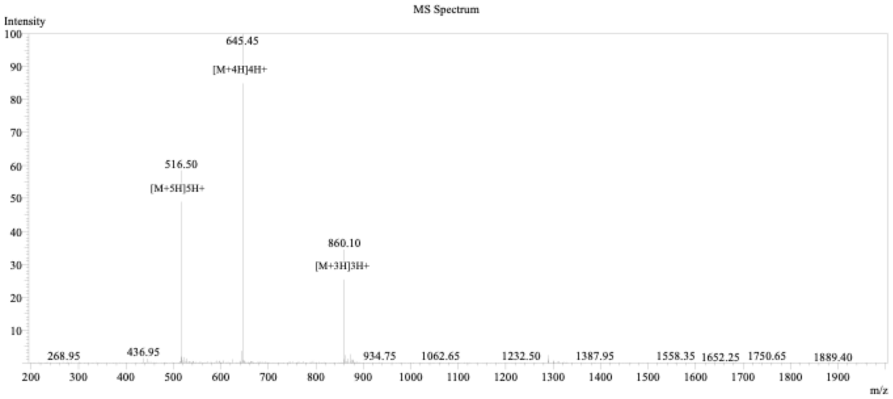

(b)

Figure S6. Analytical characterisation of synthetic T1. (a) Analytical RP-HPLC chromatogram showing the purity of T1. (b) Mass spectrum confirming the molecular mass of the synthesised peptide.

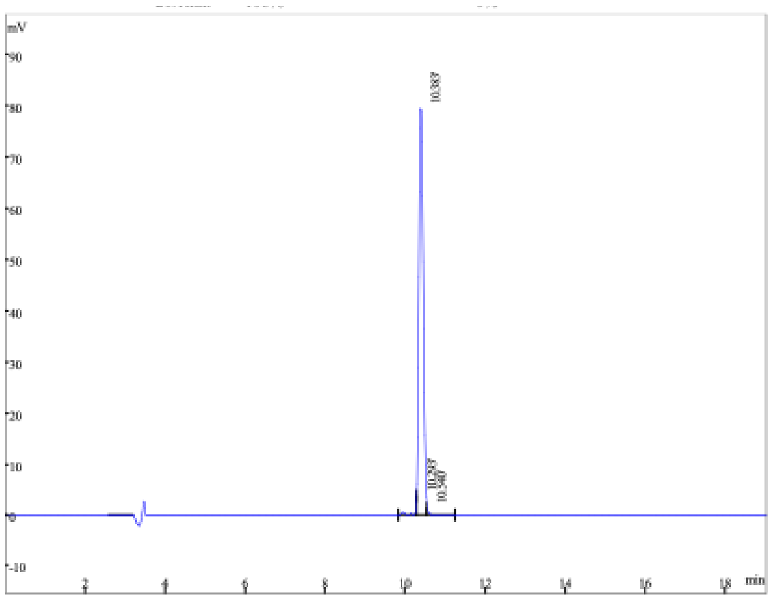

(a)

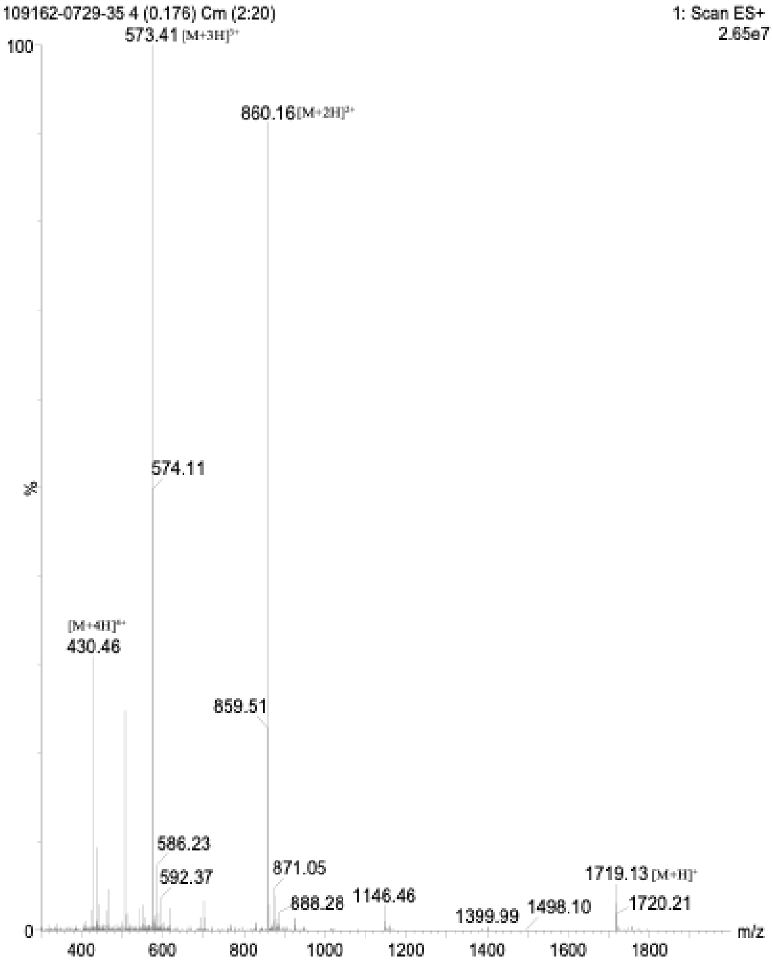

(b)

Figure S7. Analytical characterisation of synthetic T2. (a) Analytical RP-HPLC chromatogram showing the purity of T2. (b) Mass spectrum confirming the molecular mass of the synthesised peptide.

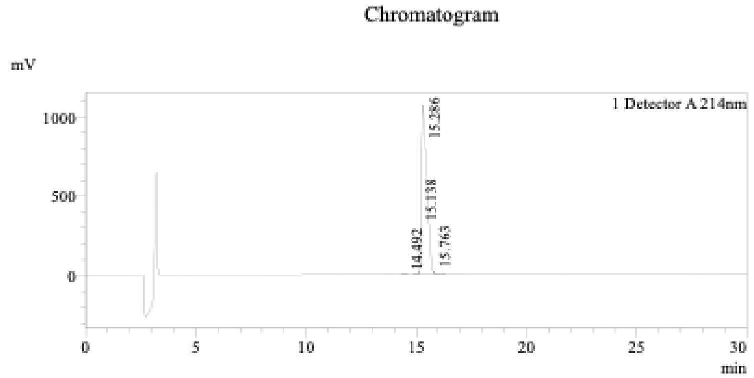

(a)

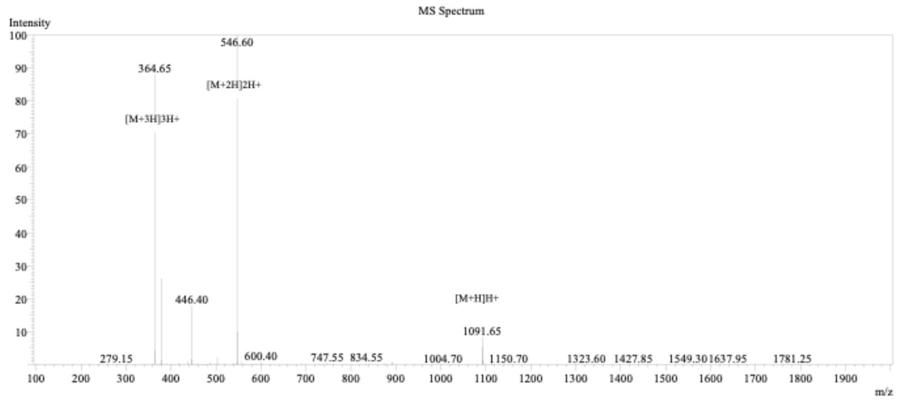

(b)

Figure S8. Analytical characterisation of synthetic T3. (a) Analytical RP-HPLC chromatogram showing the purity of T3. (b) Mass spectrum confirming the molecular mass of the synthesised peptide.

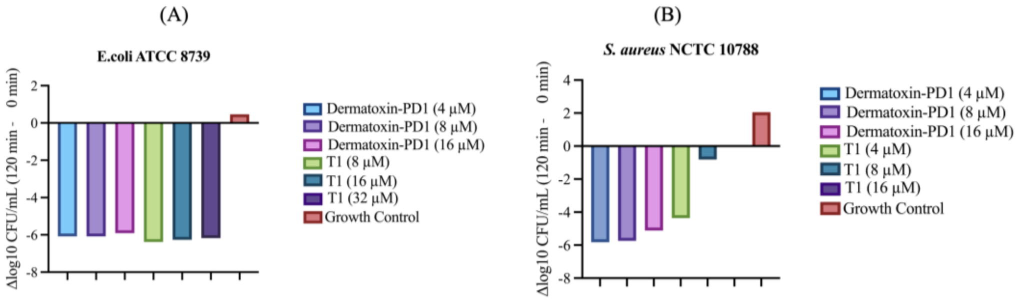

Figure S9. Changes in bacterial viability following peptide treatment, expressed as $\log_{10}$(CFU/mL) differences between 0 min and 120 min. Time-killing effects were evaluated against (A) E. coli ATCC 8739 and (B) S. aureus NCTC 10788 at the indicated peptide concentrations. Values below the detection limit were set to the detection limit for analysis. Growth controls are shown for comparison.

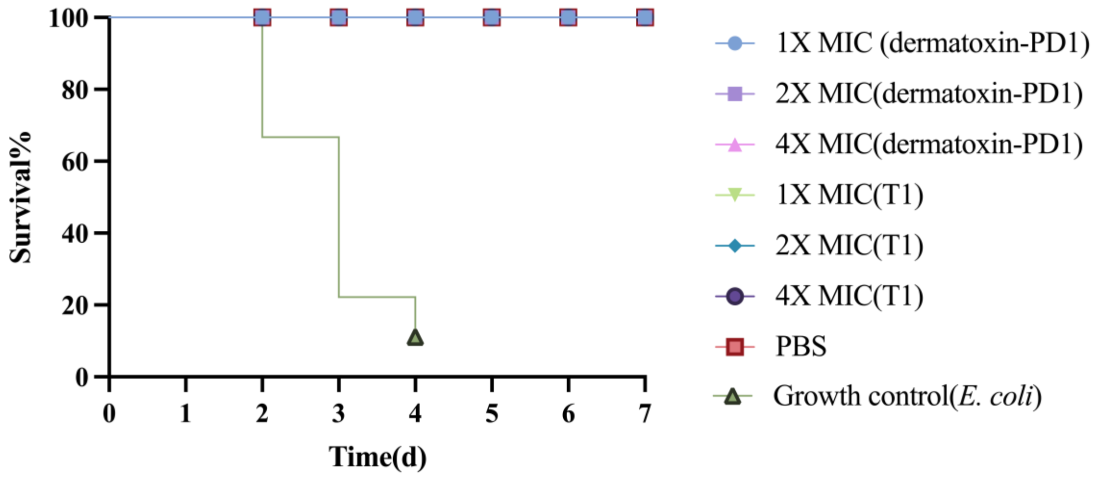

Figure S10. Survival of Galleria mellonella larvae following treatment with dermatoxin-PD1 and T1 at 1×, 2×, and 4× MIC-equivalent doses. All peptide-treated groups remained at 100% survival

throughout the observation period. Therefore, the differently coloured treatment curves are completely superimposed and appear as a single line.

1. Wechselberger, C., *Cloning of cDNAs encoding new peptides of the dermaseptin-family.* Biochimica et Biophysica Acta (BBA)-Protein Structure and Molecular Enzymology, 1998. **1388**(1): p. 279-283.
2. Amiche, M., et al., *Isolation of dermatoxin from frog skin, an antibacterial peptide encoded by a novel member of the dermaseptin genes family.* European journal of biochemistry, 2000. **267**(14): p. 4583-4592.
3. Rates, B., et al., *Peptidomic dissection of the skin secretion of Phasmahyla jandaia (Bokermann and Sazima, 1978)(Anura, Hylidae, Phyllomedusinae).* Toxicon, 2011. **57**(1): p. 35-52.
4. Chen, T., et al., *Dermatoxin and phylloxin from the waxy monkey frog, Phyllomedusa sauvagei: cloning of precursor cDNAs and structural characterization from lyophilized skin secretion.* Regulatory peptides, 2005. **129**(1-3): p. 103-108.
